# A multi-joint model of quiet, upright stance accounts for the “uncontrolled manifold” structure of joint variance

**DOI:** 10.1007/s00422-017-0733-y

**Published:** 2017-09-18

**Authors:** Hendrik Reimann, Gregor Schöner

**Affiliations:** 1 Department of Kinesiology and Applied Physiology, University of Delaware, Newark, DE USA; 2 Institute for Neural Computation, Ruhr-University, Bochum, Germany

**Keywords:** Posture, Quiet stance, Uncontrolled manifold, Modeling, Neural control, Variance

## Abstract

The upright body in quiet stance is usually modeled as a single-link inverted pendulum. This agrees with most of the relevant sensory organs being at the far end of the pendulum, i.e., the eyes and the vestibular system in the head. Movement of the body in quiet stance has often been explained in terms of the “ankle strategy,” where most movement is generated by the ankle musculature, while more proximal muscle groups are only rarely activated for faster movements or in response to perturbations, for instance, by flexing at the hips in what has been called the “hip strategy.” Recent empirical evidence, however, shows that instead of being negligible in quiet stance, the movement in the knee and hip joints is even larger on average than the movement in the ankle joints (J Neurophysiol 97:3024-3035, 2007). Moreover, there is a strong pattern of covariation between movements in the ankle, knee and hip joints in a way that most of the observed movements leave the anterior–posterior position of the whole-body center of mass (CoM) invariant, i.e., only change the configuration of the different body parts around the CoM, instead of moving the body as a whole. It is unknown, however, where this covariation between joint angles during quiet stance originates from. In this paper, we aim to answer this question using a comprehensive model of the biomechanical, muscular and neural dynamics of a quietly standing human. We explore four different possible feedback laws for the control of this multi-link pendulum in upright stance that map sensory data to motor commands. We perform simulation studies to compare the generated inter-joint covariance patterns with experimental data. We find that control laws that actively coordinate muscle activation between the different joints generate correct variance patterns, while control laws that control each joint separately do not. Different specific forms of this coordination are compatible with the data.

## Introduction

The human body in upright stance is mechanically unstable (Winter et al. 1998; Morasso and Schieppati 1999). To remain standing, the force generated by muscles along the body has to be constantly adapted by actively modulating the neural activation level based on the available sensory data. But little is known about how this mapping from sensory data to motoneural activation and ultimately to force generation is structured.

Research on quiet stance has mostly focused on the ankle joint. Analysis of the active responses to mechanical perturbation revealed that for small perturbations, most of the response occurs in the ankle musculature, while for larger perturbations, the muscle groups around the hips play a larger role (Horak and Nashner 1986; Runge et al. 1999). These response patterns have been referred to as “ankle strategy” and “hip strategy,” respectively. More recently, the ankle and hip strategies have been characterized as two modes of whole-body motion (Alexandrov et al. 2005) rather than two independent movement patterns. While less salient than the ankle mode, the hip mode is still observable even in quiet stance (Creath et al. 2005). Kiemel and colleagues have shown that the two modes might come from a single neural control strategy (Kiemel et al. 2008, 2011). Converging evidence for coordinated movements of multiple joints contributing to posture comes from the discovery of Scholz and colleagues that following a perturbation, the center of mass recovers faster and more completely than combinations of joint angles that do not affect the center of mass (Scholz et al. 2007).

Models of balance control have largely conceived of upright stance as the problem of controlling a single degree of freedom, the ankle joint (van der Kooij et al. 1999; Peterka 2002; Kiemel et al. 2002; Oie et al. 2002; Maurer and Peterka 2005; Maurer et al. 2006). This “inverted pendulum” approximation implicitly assumes that all other joints are sufficiently stiff to be passively stabilized independently of the neural controller. This is consistent with the observation that muscles converging on the ankle are correlated most strongly with postural sway (Gatev et al. 1999). It has become increasingly clear, however, that this approximation is not without problems. For one thing, there is as much movement in the knee and the hip as in the ankle joint during quiet stance (Günther et al. 2009, 2010). Moreover, different joints do not move independently of each other (Kuo 2005; Hsu et al. 2007; Pinter et al. 2008; Günther et al. 2011). In particular, Hsu et al. showed that sway patterns that leave the center of mass invariant occur more frequently than others. That observation was based on the concept of the uncontrolled manifold (UCM), a geometric representation of these sway patterns in joint space (Scholz and Schöner 1999).

But where does this structure in the covariance between the joints come from? One possibility is that the covariance is a result of active coordination between the joints by the central nervous system (CNS). This would mean that the control signal based on the sensory estimates of the body’s state in space is distributed between the different joints along the body. The specific details of this distribution would have to depend to some degree upon the current state of the peripheral neural circuitry and the configuration of the muscles and body. Another possibility is that the covariance between the joints is simply the result of the biomechanical structure of the multi-link inverted pendulum. Due to differences in inertia, modes of movement that leave the CoM invariant are inherently more excitable than those that affect the CoM (Alexandrov et al. 2005). It is conceivable that the brain simplifies the control problem by mapping sensory information about the body in space only to the ankle joint, possibly using preselected muscle synergies (Torres-Oviedo and Ting 2007), while outsourcing the stabilization of the more proximal joints to the periphery. Such a low-dimensional controller makes sense in light of the fact that most of the relevant sensory data come from the visual and vestibular systems, which provide information about the head in space, but not about the joints.

**Fig. 1:**
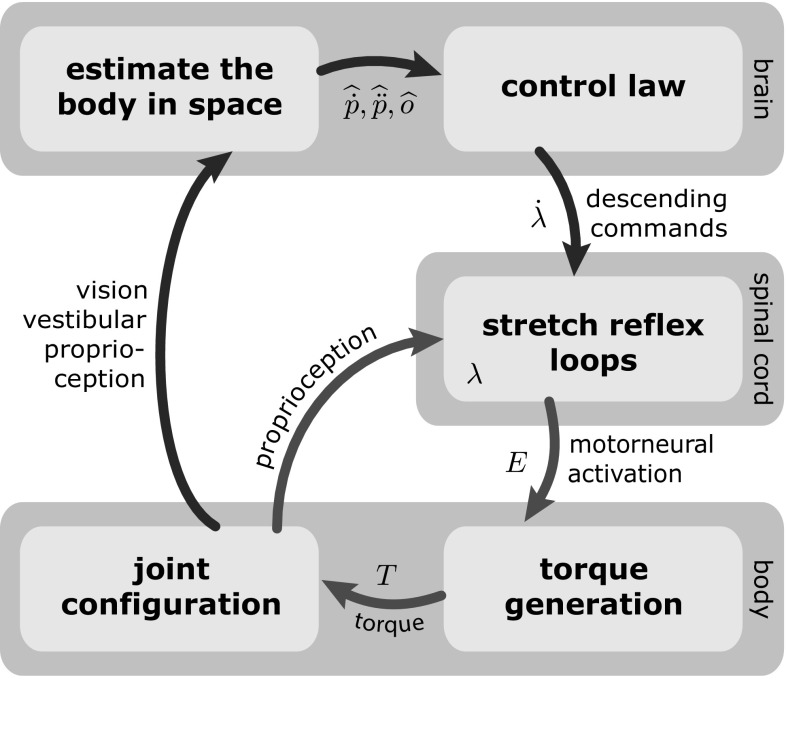
Overview of the sensorimotor loop for balancing the body in quiet, upright stance

**Fig. 2:**
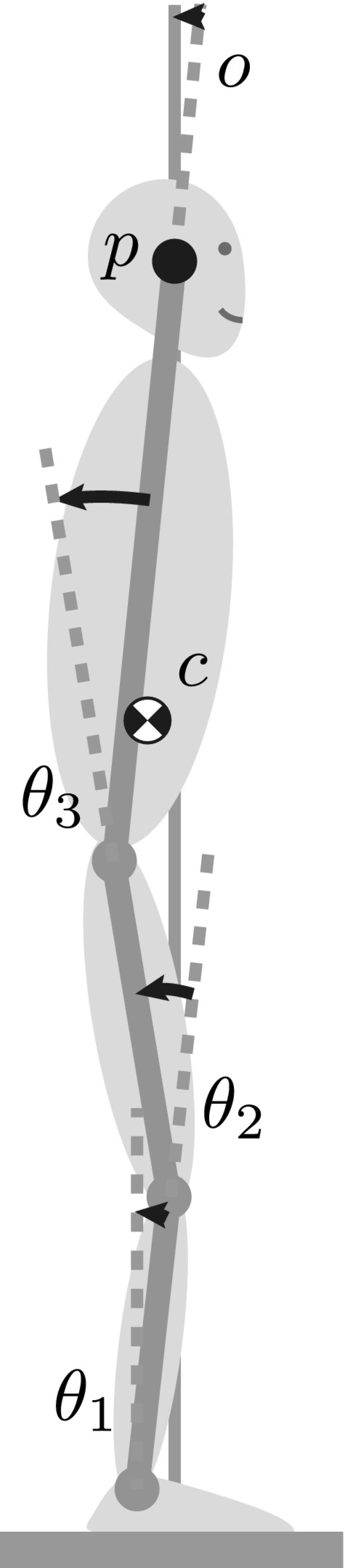
Sagittal plane model of the body in quiet, upright stance as a three-segment inverted pendulum with rotational joints at the ankle, knee and hip

In this paper, we attempt to answer this question about the source of the multi-joint covariance structure in quiet, upright stance using a detailed model of the whole sensory-motor loop of postural control as outlined in Fig. 1. The model estimates the state of the body in space from the available sensory data, generates appropriate descending motor commands, integrates these descending commands into the spinal reflex loops and takes into account the dynamics of the resulting muscle activation and of the biomechanics of the body. The body is modeled as a three-link inverted pendulum in the sagittal plane, with rotational joints at the ankle, knee and hips, as shown in Fig. 2. During quiet stance, balance in the sagittal plane is more challenging than in the frontal plane, so we restricted our analysis to this direction. For the mapping from sensory estimates of the body’s state in space to descending motor commands, we propose four different hypotheses for possible control laws with varying degrees of coordination between the joints.

## The model

Figure 1 shows the overview of the model. Most components used in the model are well established, understood and empirically validated. Each of these is briefly summarized and referenced in the following sections. We then describe the different hypotheses for the control law that maps estimates of the body’s state in space to descending motor commands.

Throughout the model section, the subscript *j* refers to the three degrees of freedom, in the order of $1 = {\textit{ankle}}$, $2 = {\textit{knee}}$ and $3 ={\textit{hip}}$. Dependencies of model terms on time, *t*, have been omitted to simplify notation where appropriate.

### The plant

In this section, we describe the model of the muscles that actuate the body and their peripheral neural control, as well as the biomechanics, that together form the plant in the narrow sense. We also characterize the sensory systems that provide estimates of the body’s state in space. All of these components are well established in the literature, although their integration in a complete model of the sensorimotor loop of upright balance control is novel.

#### Muscle model: the stretch reflex

The tonic stretch reflex activates motoneurons in response to proprioceptive information from afferent muscle spindles that are sensitive to muscle length and rate of change. Descending motor commands modulate the reflex loop by shifting the threshold length of the reflex. According to the equilibrium-point hypothesis of motor control, this modulation of threshold length is the only interaction between the higher motor areas and the spinal circuitry that generates motoneural activation and ultimately muscle contraction (Feldman 1972).

We adopt this hypothesis as an approximate description of spinal motor control (see, e.g., Raphael et al. (2010) for a more detailed model, the complexity of which was beyond the scope of our study). We further simplify the description by lumping all muscles converging on a joint into a single agonist–antagonist pair (Feldman 2011). We use the elaborated, nonlinear model of the equilibrium-point hypothesis described by Gribble et al. (1998). The activation levels of the motoneuron pools for the agonist, $E_{{\mathrm {AG}}, j}$, and antagonist, $E_{{\mathrm {AN}}, j}$, muscle groups acting on joint *j*, are determined by

1 $$\begin{aligned} E_{{\mathrm {AG}}, j}= & {} \exp \left[ \alpha _E \left( \widehat{ \theta }_j - \lambda _j + \rho _j + \mu (\widehat{\dot{\theta }}_j - \dot{\lambda }_j) \right) \right] ^+ - 1,\nonumber \\ E_{{\mathrm {AN}}, j}= & {} \exp \left[ - \alpha _E \left( \widehat{ \theta }_j - \lambda _j - \rho _j + \mu (\widehat{\dot{\theta }}_j - \dot{\lambda }_j) \right) \right] ^+ - 1.\nonumber \\ \end{aligned}$$

The half-linear function $[\cdot ]^+$ retains only the positive portion of the argument. The proprioceptive signals from afferent muscle spindles at time *t*,

2 $$\begin{aligned} \widehat{\theta }_j(t) = \theta _j(t - d_{{\mathrm {reflex}}}) + \eta _\theta , \quad \widehat{\dot{\theta }}_j(t) = \dot{\theta }_j(t - d_{{\mathrm {reflex}}}) + \eta _{\dot{\theta }},\nonumber \\ \end{aligned}$$

are delayed, noisy copies of the joint angle, $\theta _j$, and joint velocity, $\dot{\theta }_j$, where $d_{{\mathrm {reflex}}}$ is a time delay and $\eta _*$ is time-correlated noise modeling the variability of neural processing (see below). The parameters $\lambda _j$ and $\dot{\lambda }_j$ are neural representations of the stretch reflex threshold length and its rate of change, and $\rho _j$ determines the co-contraction of the agonist–antagonist muscle pair. We assume that during quiet stance, the threshold length $\lambda _j$ is represented locally in the spinal cord, while its rate of change $\dot{\lambda }_j$ is determined in the brain and sent to the spinal cord as a descending motor command (see Fig. 1) and that the co-contraction command, $\rho _j$, is constant and small. For the model, this implies that $\dot{\lambda }_j$ is the input variable for the spinal reflex loops, while $\lambda _j$ is determined by numerical integration of the stochastic signal $\dot{\lambda }_j$ (see Sect. 2.3). The velocity dependence of the stretch reflex is modeled as relative to the rate of change, $\dot{\lambda }_j$, of the threshold length (Lussanet et al. 2002). The values of the constant parameters $\alpha _E$ and $\mu$ were adapted from Laboissière et al. (1996) and Micheau et al. (2003).

The total motoneural activation, $E_j$, for the lumped agonist–antagonist pair at the *j*th joint is the sum

3 $$\begin{aligned} E_j = \left( - E_{{\mathrm {AG}}, j} + E_{{\mathrm {AN}}, j} \right) \eta _m, \end{aligned}$$

where $\eta _m$ is multiplicative white noise with mean $\mu _m = 1$ (Faisal et al. 2008).

To account for the physical limits of force generation in muscles, we introduced a boundary, $E_{\mathrm {max}}$, for the motoneuron activation, $E_{{\mathrm {AG}}}$ and $E_{{\mathrm {AN}}}$. Beyond the point where the combined activation of the proprioceptive pathways pushes the motoneuron activation to within 75% of that boundary, we modeled the motor neuron activation through a hyperbolic function that is shallower than the exponential function used outside that region (Eq. 1).

4 $$\begin{aligned} \widetilde{E}_{{\mathrm {AG/AN}}} = c_1 (E_{{\mathrm {AG/AN}}} + c_2)^{-1} + c_3. \end{aligned}$$

We chose the parameters, $c_i$, such that this function converges to $E_{{\mathrm {max}}}$ for large activation levels and connects smoothly to the exponential function at the crossover levels of activation. The maximal motoneuron activation was set to $E_{{\mathrm {max}}} = 10$, which results in a maximal ankle torque similar to the maximal isometric ankle torques observed in humans (Hasson et al. 2011). Because the regime of very large activation levels was never reached during simulations of quiet stance, this modification of the model at high activation levels served only to test hypothetical extreme cases in which we pushed the system toward falling (see Sect. 4). In these cases, limiting the maximal activation level made it harder for the model to remain upright.

#### Muscle model: torque generation

Muscle–tendon complexes generate joint torque both from active contraction and from passive viscoelastic properties. Given a level of motoneural activation, *E*, the torque generated by muscle contraction at a joint depends on the size of the muscle, described by the physiological cross-sectional area (PCSA), and on the moment arm that transforms force into torque. We capture these two characteristics by the muscle distribution matrix, *A*, that relates the vector of motoneural activation for each joint, *E*, to the vector of steady-state active joint torques,

5 $$\begin{aligned} \widetilde{T}_{{\mathrm {act}}} = A E. \end{aligned}$$

Several important muscles in the leg, such as the gastrocnemius and the hamstrings, are biarticular. This plays an important role for the stiffness characteristics of a musculoskeletal system (Franklin and Milner 2003) and is incorporated into our model by the off-diagonal elements in the muscle distribution matrix, *A* (Rozendaal and Van Soest 2008). We used values for the PCSA provided by Ward et al. (2009) and moment arms from Van Soest et al. (2003) to estimate this matrix of parameters as

6 $$\begin{aligned} A =\begin{pmatrix} 10.94 &{}\quad 1.1 &{}\quad 0 \\ 0 &{}\quad 7.43 &{}\quad 1.2 \\ 0 &{}\quad 0.94 &{}\quad 9.10 \end{pmatrix}\,{\mathrm {N\,m}}. \end{aligned}$$

The instantaneous torque, $T_{{\mathrm {act}}}$, lags behind the steady-state torque $\widetilde{T}_{{\mathrm {act}}}$ due to calcium kinetics. Following Gribble et al. (1998), we model the time course of torque generation as a critically damped second-order low-pass filter,

7 $$\begin{aligned} \tau _m^2 \ddot{T}_{{\mathrm {act}}} + 2 \tau _m \dot{T}_{{\mathrm {act}}} + T_{{\mathrm {act}}} = \widetilde{T}_{{\mathrm {act}}}, \end{aligned}$$

with relaxation time, $\tau _m = 15\, {\mathrm {ms}}$.

**Table 1:** All parameter values used for the simulation experiments

$d_{{\mathrm {reflex}}}$	$30\,{\mathrm {ms}}$	Spinal stretch reflex time delay
$d_{{\mathrm {brain}}}$	$120\,{\mathrm {ms}}$	Time delay for sensory estimation in the brain
$\alpha _E$	$12\,{\mathrm {rad}}^{-1}$	Stretch reflex form parameter
$\rho$	$0.01\,{\mathrm {rad}}$	Co-contraction command
$\mu$	$0.1\,{\mathrm {s}}$	Stretch reflex velocity gain
$\tau _m$	$15\,{\mathrm {ms}}$	Time constant of the calcium kinetics low-pass filter
$\alpha _\eta$	$5\,{\mathrm {s^{-1}}}$	Ornstein–Uhlenbeck process inverse correlation time
$\sigma _{\theta }$	$0.002\,{\mathrm {rad}}$	Muscle spindle activation noise (position)
$\sigma _{\dot{\theta }}$	$0.005\,{\mathrm {rad\,s}}^{-1}$	Muscle spindle activation noise strength (velocity)
$\sigma _{\dot{p}}$	$0.007 / 0.01\,{\mathrm {m\,s}}^{-1}$	Head velocity estimation noise strength (EO/EC)
$\sigma _{\ddot{p}}$	$0.03 / 0.032\,{\mathrm {m\,s}}^{-2}$	Head acceleration estimation noise strength (EO/EC)
$\sigma _{o}$	$0.025 / 0.032\,{\mathrm {rad}}$	Trunk orientation estimation noise strength (EO/EC)
$\sigma _{\dot{\lambda }}$	$0.001\,{\mathrm {rad\,s}}^{-1}$	Neural processing noise
$\sigma _m$	$0.01\,{\mathrm {rad}}$	Signal-dependent motor noise
$\alpha _{\dot{p}}$	$12\,{\mathrm {rad}}^{-1}\,\mathrm{s}^{-2}$	Head velocity gain
$\alpha _{o}$	$40 \,{\mathrm {rad}}^{-1}\,\mathrm{s}^{-3}$	Trunk orientation gain

The contributions of the passive elastic properties of the muscle-tendon complex to joint torques were modeled as sums of exponentials

8 $$\begin{aligned} T_{{\mathrm {ela}}, j} = e^{ a_{j0} + \sum a_{ji} \theta _i } - e^{b_{j0} + \sum b_{ji} \theta _i} + c_{ji}, \end{aligned}$$

following Riener and Edrich (1999), where $a_{ji}, b_{ji}$ and $c_{ji}$ model how the passive torque at the *j*th joint depends on the state of the *i*-th joint. For the knee joint, an additional exponential term accounts for the steep increase in torque when the knee is fully extended. The passive viscous properties of muscles and joints were modeled as a simple linear damper element, in vector notation

9 $$\begin{aligned} T_{{\mathrm {vis}}} = - B \dot{\theta }, \end{aligned}$$

where the matrix of parameters

10 $$\begin{aligned} B =\begin{pmatrix} 25 &{}\quad 2.51 &{}\quad 0 \\ 0 &{}\quad 16.98 &{}\quad 2.74 \\ 0 &{}\quad 2.15 &{}\quad 20.80 \end{pmatrix} {\mathrm {N\,m\,s\,rad^{-1}}} \end{aligned}$$

was chosen to be proportional to the muscle distribution matrix *A*. Because damping comes primarily from muscle properties through their force–velocity characteristic, we assumed that damping is distributed across joints and muscles analogously to how stiffness is (as modeled by the stiffness matrix *A*).

The total instantaneous torque vector, *T*, generated by the muscles and tendons is the sum

11 $$\begin{aligned} T = T_{{\mathrm {act}}} + T_{{\mathrm {ela}}} + T_{{\mathrm {vis}}} \end{aligned}$$

of active contraction and passive viscoelastic contributions.

#### Biomechanical dynamics

We model the body as a three-link inverted pendulum in the sagittal plane, with joints at the ankle, knee and hip, as illustrated in Fig. 2. Using the joint angles, $\theta _j$, as generalized coordinates, the equation of motion is given in vector notation as

12 $$\begin{aligned} M(\theta ) \ddot{\theta }+ C(\theta , \dot{\theta }) \dot{\theta }+ N(\theta ) = T, \end{aligned}$$

where *M* is the inertia matrix, *C* represents the Coriolis and centrifugal forces, *N* is the vector of gravitational forces and *T* is the vector of external torques generated by muscles and tendons. The dynamic quantities $M(\theta )$, $C(\theta , \dot{\theta })$ and $N(\theta )$ were determined by Lagrangian dynamics using screw theory (Murray et al. 1994). The required anthropometric data were estimated based on body height $h = 1.80\,{\mathrm {m}}$ and mass $m = 80\,{\mathrm {kg}}$ using standard techniques (Winter 1990).

The forward kinematic equations specify the anterior–posterior position, *p*, of a point on the upper segment that represents the head position in space. The orientation, *o*, of the trunk segment around the medial–lateral axis can also be calculated. Taking the derivative with respect to time yields formulas for the velocity, $\dot{p}$, and acceleration, $\ddot{p}$, of the head.

#### Sensory signals estimating the kinematic state of the body in space

Different sensory systems provide information about the kinematic state of the body in space, most notably vision, the vestibular system and proprioception. We assume that the CNS combines these sensory streams to form estimates of different variables that are relevant to the stabilization of upright stance, in particular, the velocity, $\dot{p}$, and acceleration, $\ddot{p}$, of the anterior–posterior head position, *p*, and the orientation, *o*, of the trunk around the medial–lateral axis with respect to the vertical. These estimates are modeled as:

13 $$\begin{aligned} \widehat{\dot{p}}(t)= & {} \dot{p}(t-d_{{\mathrm {brain}}}) + \eta _{\dot{p}},\nonumber \\ \widehat{\ddot{p}}(t)= & {} \ddot{p}(t-d_{{\mathrm {brain}}}) + \eta _{\ddot{p}},\nonumber \\ \widehat{o}(t)= & {} o(t-d_{{\mathrm {brain}}}) + \eta _{o}. \end{aligned}$$

The delay parameter, $d_{{\mathrm {brain}}}$, accounts for the processing time required to form these neural estimates. Neural processing noise is modeled as time-correlated noise, $\eta _*$.

We model two different sensory conditions, eyes open (EO) and eyes closed (EC). As we assumed vision to be one of the sensory modes feeding into the estimate of the kinematic state of the body in space, we interpreted the removal of vision as a decrease in accuracy of state estimation. We modeled this by increasing the magnitude of the sensory noise, $\eta _{\dot{p}}, \eta _{\ddot{p}}$ and $\eta _{o}$ (see Table 1 at the end of this section for details).

### Control law

The brain controls muscle activation by modulating the thresholds, $\lambda$, of the activation laws of each muscle–joint system (Eq. 1). To stabilize upright stance, the brain must shift the threshold lengths of all joints according to a control law that takes the sensed deviations from the upright state (Eq. 13) as input. This control law must transform signals about the body in space to descending motor commands.

We build a model of this control law in two steps. We first postulate feedback terms, *f*, based on sensory estimates of the body’s state in space designed to stabilize the body in space. This follows models used in the literature that do not address multiple degrees of freedom (Peterka 2002). The second step is to transform these feedback terms into descending motor commands, $\dot{\lambda }$, that modulate the spinal reflex loops. The critical element in this step is the synergistic forward network mapping the low-dimensional feedback to multiple degrees of freedom.

#### Controlling the body in space

A control law stabilizing the body in space generates control signals at the level of the variables $\dot{p}$, $\ddot{p}$ and *o*. We define feedback terms, $f_p$ and $f_o$, for control laws at this level based on the sensory estimates, $\widehat{\dot{p}}, \widehat{\ddot{p}}$ and $\widehat{o}$, estimating the kinematic state of the body in space (Eq. 13),

14 $$\begin{aligned} f_p = -\alpha _{\dot{p}} \widehat{\dot{p}} -\alpha _{\ddot{p}} \widehat{\ddot{p}}, \quad f_o = - \alpha _o \widehat{o} \in {\mathbb {R}}, \end{aligned}$$

where the constants, $\alpha _*$, are gain parameters. The acceleration gain was chosen to depend upon the velocity gain as $\alpha _{\ddot{p}} = 2 \zeta \sqrt{\alpha _{\dot{p}}}$ with a damping ratio of $\zeta = 0.5$.

These feedback signals could be used to create a damped harmonic oscillator for the head movement state, $\dot{p}$. For instance, in the absence of noise, processing delays and other perturbations, setting $\dddot{p} = f_p$ would regulate the head movement to $\dot{p} = 0$, stopping all movement.^1^ Inverted pendulum models of postural control that neglect muscle dynamics directly use such feedback signals to specify torques at the level of the ankle joint (e.g., van der Kooij et al. 1999; Peterka 2002; Kiemel et al. 2002; Oie et al. 2002; Maurer and Peterka 2005; Maurer et al. 2006), usually including proportional and derivative terms.

#### Transformation into descending motor commands

The challenge here is that the available sensory estimates about the body in space and the feedback terms based on these are one-dimensional, while the descending motor commands are multi-dimensional, with one component each for the ankle, knee and hip joints. In the following, we develop four hypotheses for how this one-to-many mapping might be structured.

***A.*** *Ankle strategy with co-contraction at proximal joints*

This control scheme assumes an ankle strategy, mapping the body-in-space feedback to the ankle joint, setting $\dot{\lambda }_1 = f_p$. Stability at the knee and hip joints is achieved by increasing the local stiffness via co-contraction of the relevant muscles, modeled by setting the co-contraction parameter to $\rho _{2, 3} = 0.15\,{{\mathrm {rad}}}$ and assuming a constant activation threshold parameter, i.e., $\dot{\lambda }_{2, 3} = 0$.

***B.*** *Ankle strategy with local feedback control of proximal joints*

In this second control scheme, we assume that the spinal reflex loops at each joint are actively modulated based on proprioceptive feedback about the state of that joint, but still not integrated into multi-joint coordination.

15 $$\begin{aligned} \dot{\lambda }_j = - \alpha _\theta ( \widehat{\theta }_j - \theta ^{\mathrm {(ref)}}_j ) - \alpha _{\dot{\theta }} \dot{\theta }_j \end{aligned}$$

for $j = 2, 3$, where $\alpha _*$ are gain parameters and $\theta ^{{\mathrm {(ref)}}}_j$ is a joint angle reference. For the ankle joint, the body-in-space feedback is added to this local feedback,

16 $$\begin{aligned} \dot{\lambda }_1 = - \alpha _\theta ( \widehat{\theta }_1 - \theta ^{{\mathrm {(ref)}}}_1 ) - \alpha _{\dot{\theta }} \dot{\theta }_1 + f_p. \end{aligned}$$

***C.*** *Ankle strategy with multi-joint coordination*

The third control approach incorporates the ankle strategy into a coordination scheme. The first step is to transform the body-in-space feedback into a multi-dimensional signal, which we do by setting

17 $$\begin{aligned} F_p = \tilde{J_p}^+ f_p \in {\mathbb {R}}^3, \end{aligned}$$

where $$\tilde{J_p} = \begin{pmatrix} \frac{\partial p}{\partial \theta _1}&\quad 0&\quad 0 \end{pmatrix}$$ is the Jacobian matrix relating changes in the ankle joint angle, $\theta _1$, to changes in *p*, and the $(~)^+$ indicates the Moore–Penrose pseudo-inverse (Siciliano and Khatib 2008). The additional dimensions allow us to also incorporate trunk orientation feedback without affecting the center of mass. We do this by defining the joint-level orientation feedback as

18 $$\begin{aligned} F_{o} = \tilde{J}_o^{+} \begin{pmatrix} f_o \\ 0 \end{pmatrix} \in {\mathbb {R}}^3, \end{aligned}$$

where

19 $$\begin{aligned} \tilde{J}_o = \begin{pmatrix} J_o \\ J_c \end{pmatrix} \in {\mathbb {R}}^{2 \times 3} \end{aligned}$$

is the augmented Jacobian for the given constraint (Siciliano 1990) and $J_c, J_o \in {\mathbb {R}}^{1 \times 3}$ are the Jacobians of the whole-body center of mass, *c*, and the trunk orientation, *o*. The joint-level feedback terms from head position and trunk orientation are integrated by simple summation

20 $$\begin{aligned} F = F_p + F_o \end{aligned}$$

into a combined kinematic joint-level feedback term *F* for both sub-tasks.

The second step is to transform kinematic joint-level feedback, *F*, into descending motor commands, $\dot{\lambda }$. We assume that the CNS has learned to modulate the feedback commands to the muscle–joint systems to compensate for the inertia of the linked body segments and the viscoelastic properties of the muscles and is able to modulate the feedback command to account for them.

21 $$\begin{aligned} \dot{\lambda }= R^{-1} A^{-1} M F + \eta _{\dot{\lambda }} \in {\mathbb {R}}^3. \end{aligned}$$

Multiplying the feedback signal with the inertia matrix, *M*, amounts to assuming that joints facing larger inertial moments receive larger motor commands. Multiplication with the inverse of the muscle distribution matrix, *A*, amounts to assuming that muscles that are more effective in generating joint torques (due to their lever arm or their size) receive less activation. The matrix

22 $$\begin{aligned} R = R(\widehat{\theta }, \widehat{\dot{\theta }}, \lambda , \dot{\lambda }) = \frac{\partial E(\widehat{\theta }, \widehat{\dot{\theta }}, \lambda , \dot{\lambda })}{\partial \lambda } \end{aligned}$$

relates changes of the activation threshold, $\lambda$, to changes of the motoneural activation, *E*. Multiplying with its inverse ensures that muscles that are in a steep portion of their activation characteristic receive a weaker command than muscles that are in a flatter portion of their activation characteristic. The term $\eta _{\dot{\lambda }}$ represents neural processing noise in the descending signals.

By using the inertia matrix, *M*, in Eq. 21, we assume that the CNS has an internal model about the distribution of mass in the current body configuration. This is a reasonable assumption, because in normal movement generation, interaction torques are usually canceled out almost perfectly with essentially no time delay (Winter 1995). In contrast, we do *not* assume an internal model of the gravitational torques acting on each joint. This would be a much stronger assumption, which is not necessary, as shown by our results. Using *A* and *R* in the control law assumes that the CNS knows the distribution of muscles along the body, which is a constant, and the current neural activation, which amounts to assuming that an efference copy of the motor command is available.

***D.*** *Distributed strategy with multi-joint coordination*

The last control scheme we analyze also employs multi-joint coordination, but instead of mapping the body-in-space feedback only to the ankle joint, it is distributed among all available joints according to a principle of minimum intervention in joint space (Todorov and Jordan 2002). This implies that the joint-level feedback, $F_p$, used to enact a desired feedback on the body-in-space level, $f_p$, should be as small as possible in joint space, which is achieved by using the least-squares pseudo-inverse solution of the full Jacobian, setting

23 $$\begin{aligned} F_p = J_p^+ f_p \in {\mathbb {R}}^3. \end{aligned}$$

The orientation feedback term, $F_o$, and transformation from joint-level feedback to descending motor commands are the same as defined in the previous control scheme (Eqs. 18 and 21).

### Modeling neural processing noise

To probe the stability of the modeled process, we have added noise terms at different levels. These are meant to capture neural processing noise. Fluctuations in neural populations are characterized by spatiotemporal correlations captured by Ornstein–Uhlenbeck processes (Smith 2010; Ricciardi and Sacerdote 1979; Lánský and Sacerdote 2001). Mathematically, an Ornstein-Uhlenbeck process can be obtained by numerically solving the stochastic differential equation

24 $$\begin{aligned} \dot{\eta }_t = - \alpha _\eta \eta _t + \xi \end{aligned}$$

in time, where $\eta$ is the resulting time-correlated noise, $1/\alpha _\eta$ is its correlation time and $\xi$ is Gaussian white noise. We modulate the noise magnitude by scaling the variance of $\xi _*$ to a parameter $\sigma _*$, where the asterisk denotes the different noise terms.

### Parameter values

The model parameters describing muscle physiology and the stretch reflexes in the spinal cord are constrained by the experimental literature. Some parameter settings represent simplifications to avoid the model becoming overly complex, e.g., a single value $d_{{\mathrm {reflex}}}$ for the delay of proprioceptive feedback at the ankle, knee and hip joints, although the actual time delays are different (Latash 1993).

The free parameters that could be adjusted to fit the statistical properties of the sway trajectories were the feedback gains $\alpha _{\dot{p}}, \alpha _{\ddot{p}}$ and $\alpha _o$ and the noise magnitudes $\sigma _{\theta }, \sigma _{\dot{\theta }}, \sigma _{\dot{p}}, \sigma _{\ddot{p}}, \sigma _{o}, \sigma _{\dot{\lambda }}$ and $\sigma _{m}$. These parameters were tuned by hand to reproduce the geometrical and temporal characteristics of experimental sway trajectories in quiet stance. The tuning process consisted of an iterative grid search approach, where we started with a grid that covered the physiologically feasible range for each parameter, simulated the model with this parameter set with $N = 6$ repetitions. Then we compared the resulting variance measures $V_{\Vert }$ and $V_{\perp }$ in the uncontrolled manifold (UCM) basis of the CoM, head position and trunk orientation as described in “Methods” section with the experimental results and picked the parameters settings with the best fit. This process was repeated until either the simulation results were roughly similar to the experimental data, or until the grid search yielded no options to markedly improve the fit quality. The resulting parameter set was used as the best fit for each control approach and simulated $N=48$ times in the simulation study (see Sect. 3.1 below). The resulting parameter values are summarized in Table 1.

## Methods

Our results are based on simulations that capture qualitative properties of the model and detailed, quantitative comparisons to experimental data.

### Simulations

The mathematical model was simulated in MATLAB by solving the set of stochastic differential equations numerically. We employed the stochastic Euler method with a time step of $2\,{\mathrm {ms}}$. The initial kinematic state of the body was taken from Van Soest et al. (2003) as

25 $$\begin{aligned} \theta ^{(0)} = \begin{pmatrix} -0.1 \\ 0.2 \\ -0.2 \end{pmatrix}, \quad \dot{\theta }^{(0)} = 0. \end{aligned}$$

This resulted in an initial center of mass position of $c \approx 3\,{\mathrm {cm}}$ anterior to the ankle joint. The initial values of the threshold parameters $\lambda$ were chosen such that the sum of passive and active torques exactly canceled out the gravitational torques at each joint. For a fixed $\theta$, the force–length relationship (Eq. 1) is monotonic in $\lambda$ and thus invertible, so $\lambda ^{(0)}$ is uniquely determined by Eq. 25 and the initial constraint $\ddot{\theta }^{(0)} = 0$ and can be calculated as

26 $$\begin{aligned} \lambda _j = \left\{ \begin{array}{ll} \frac{{{\mathrm{\mathrm {sgn}}}}E_j}{\alpha _E} \log \bigl ( |E_j| + 1 \bigr ) + \theta _j - {{\mathrm{\mathrm {sgn}}}}E_j \rho &{} : |E_j| > e^{2 \alpha _E \rho } - 1 \\ \frac{1}{\alpha _E}{{\mathrm{\mathrm {asinh}}}}\bigl ( \frac{E_j}{2} e^{- \alpha _E \rho } \bigr ) \frac{1}{\alpha _E} + \theta _j &{} : \qquad {\mathrm {else.}} \end{array}\right. \nonumber \\ \end{aligned}$$

The initial conditions of all sensor estimates were set to the actual values of the estimated variables. All variables were assumed to be constant for $t < 0$. The first $5\,{\mathrm {s}}$ of each trial were disregarded to avoid possible artifacts from these fixed initial settings.

For each of the four control hypotheses described in Sect. 2.2.2, we simulated $N = 48$ trials. For the versions using co-contraction and local feedback control, we had to remove the neural processing delays in Eqs. 2 and 13, because these strategies failed to enable upright stance in the presence of delay (so clearly, these alternatives are not viable, but we want to see their effect on the UCM structure of variance).

### Empirical data

To compare the model to empirical data, we reanalyzed an experimental data set of quiet stance in which ten human participants stood upright with their arms folded on a normal support surface for 5 min (Hsu et al. 2007), either with eyes open (EO) or closed (EC). Nine infrared markers with 1 cm diameter were attached to the subjects’ body (for details, please refer to Hsu et al. (2007)). The marker positions were recorded using a VICON optical motion measurement system (Oxford Metrics) at $120\,{\mathrm {Hz}}$. For comparison with the model, we transformed the marker data into joint angles for the ankle, knee and hip joints.

The present model encompasses stabilizing feedback on a short and medium time scale. Drifts over a long time scale might still lead to configurations that are unstable. We assume that the CNS has additional mechanisms to identify and counter these slow drifts, but these are not part of the current study. For that reason, we partitioned the experimental data into shorter episodes of 30 s each. Each trial yielded 8 such episodes, the first one starting 10 s after trial start and each subsequent one starting where the previous one stopped. This resulted in a total of 240 episodes from 10 subjects with 3 trials each, both for the EO and the EC condition.

### Data analysis

We analyzed the structure of sway variance in joint space of both the experimental and the simulated joint trajectories in quiet stance using the uncontrolled manifold (UCM) approach (Scholz and Schöner 1999). The UCM is a statistical tool for the hypothesis-based analysis of variance in a multi-dimensional data set. It is based on the idea that the CNS stabilizes those aspects of the motor system that are relevant for a given task, while leaving other aspects comparatively free. For instance, in a reaching movement, the CNS would monitor and control the position of the hand more strictly than the position of the elbow. High levels of control are associated with low levels of variance, so this idea of selective stabilization implies that the variance of task-relevant variables is low relative to the variance of task-irrelevant variables.

This notion leads to testable hypotheses about the structure of the covariance between different degrees of freedom (DoF) of the motor system in repetitive tasks. If it is true, then the task-relevant variance is expected to be significantly smaller than the task-irrelevant variance. The task-irrelevant variance can be defined as the variance *parallel to* the UCM, corresponding to the movements that *do not* affect the task variable. Similarly, the task-relevant variance is defined as the variance *orthogonal to* the UCM, corresponding to the movements that *do* affect the task variable.

We refer to variance parallel to the UCM as $V_\Vert$ and to variance orthogonal to the UCM as $V_\perp$. Formally, these two magnitudes can be estimated by calculating the projections of the sample covariance matrix $\widehat{\Sigma }$ onto the null space of the task Jacobian *J* and its orthogonal complement. The projection matrices $E_\Vert$ and $E_\perp$ can be obtained by a singular value decomposition of *J*. The magnitude of the variance in each subspace is estimated by the traces

27 $$\begin{aligned} V_\Vert = \frac{1}{k_\Vert } {{\mathrm{\mathrm {tr}}}}\left( E_\Vert ^T \widehat{\Sigma }E_\Vert \right) \quad {\mathrm {and}} \quad V_\perp = \frac{1}{k_\perp } {{\mathrm{\mathrm {tr}}}}\left( E_\perp ^T \widehat{\Sigma }E_\perp \right) \nonumber \\ \end{aligned}$$

of the projected sample covariance matrices, normalized by the dimension $k_\Vert , k_\perp$ of the subspaces. A more detailed and technically refined description of how to calculate these measures has recently been presented in Yen and Chang (2010). For statistical analysis, the variance measures were log-transformed (Verrel 2010). As first stated by Müller and Sternad (2003) and analyzed in detail by Verrel (2011), UCM effects can result both from covariation between elemental variables and from differences in their marginal variances (see also commentary by Schöner and Scholz (2007). To disambiguate these two sources of structure, we also analyzed a decorrelated data set, using the randomization method (Müller and Sternad 2003).

## Results

We first present a comparison of the different control hypotheses. In the following, we focus on hypothesis (D) using distributed control with multi-joint coordination. All results presented in Sect. 4.3 and later are from this version.

### Standing upright

**Fig. 3:**
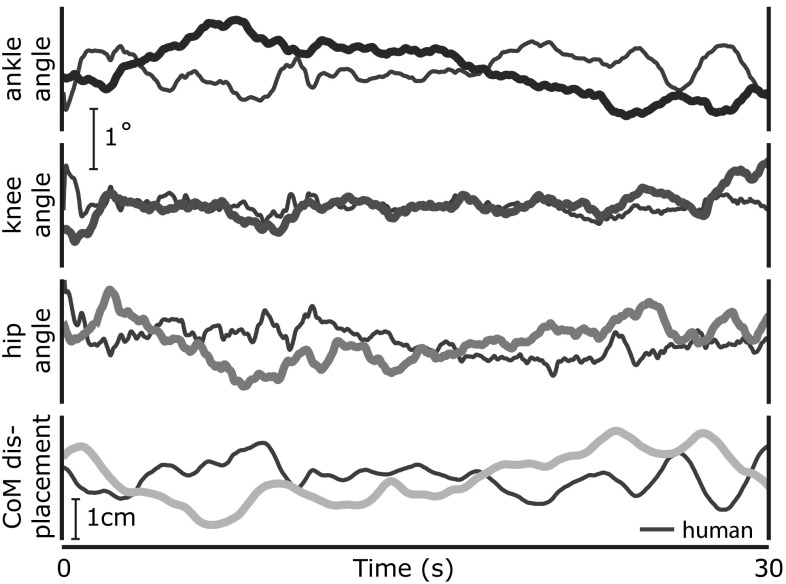
Example trajectories of the joint angles and anterior–posterior center of mass position from one model simulation (*colored*) and one human trial (*gray*), using control scheme *D*

The model was capable of stabilizing the simulated body against fluctuations from sensory estimation errors, neural processing noise and the destabilizing effects of gravity. Figure 3 illustrates this in a sample simulation showing trajectories of the joint angles and the anterior–posterior CoM in the eyes open condition. Sample trajectories from a single human trial (EO) are also presented for visual comparison. In this example, control scheme *D* was used for the transformation into descending motor commands (c. Sect. 2.2.2).

**Fig. 4:**
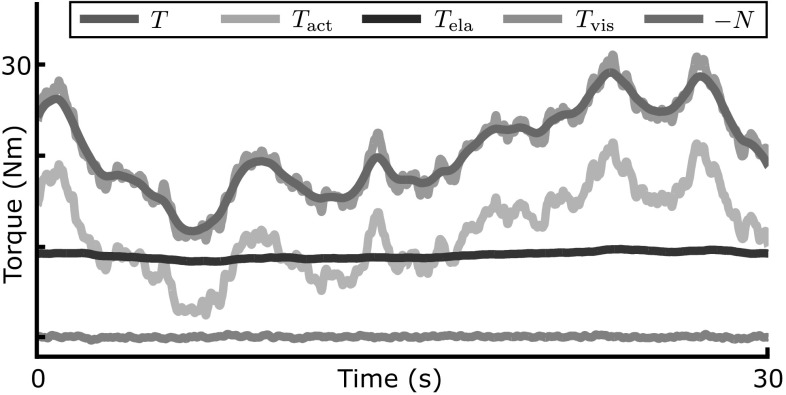
Trajectories of the torques acting on the ankle joint from the same model simulation as in Fig. 3. The total torque *T* from the muscle-tendon system is the sum of torque from active muscle contraction ($T_{{\mathrm {act}}}$) and passive elastic and viscous ($T_{{\mathrm {ela}}}, T_\mathrm {vis}$) torques

Figure 4 shows the time course of the torque at the ankle joint from the same simulation. The ankle torque is separated into active torque, $T_{{\mathrm {act}}}$, generated by muscle contraction, and passive torques, $T_{{\mathrm {ela}}}$ and $T_{{\mathrm {vis}}}$, that arise from the elastic and viscous properties of the muscle–joint system. The gravitational torque $-N$ is also plotted, its sign inverted for easier comparison. The sample trajectories illustrate that the level of passive elastic torque is substantial and sometimes exceeds the magnitude of the active torque, but its modulation in time is minimal.

**Fig. 5:**
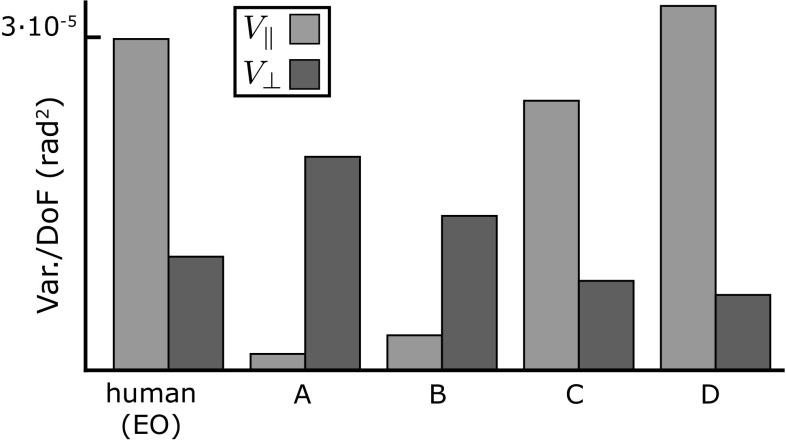
Results of the UCM analysis with respect to the anterior–posterior CoM for the human data (*left*) and the model simulations using four different control hypotheses. **a** Ankle strategy with co-contraction at proximal joints. **b** Ankle strategy with local feedback control of proximal joints. **c** Ankle strategy with multi-joint coordination. **d** Distributed strategy with multi-joint coordination

### Comparison between the control schemes

To analyze the geometrical structure of the sway patterns in the three-dimensional joint space spanned by the ankle, knee, and hip angles, we performed UCM analysis with respect to the anterior–posterior CoM position for the data generated by the model with each of the four different control hypotheses. Figure 5 shows the results in comparison to experimental data. Both the ankle strategy with co-contraction (A) and with local feedback (B) result in covariance patterns where there is substantially more variation in joint angle combinations that affect the CoM ($V_\perp$) than in directions that leave the CoM invariant ($V_\Vert$). This changes radically with the introduction of active coordination between the joints. For both the ankle strategy with active coordination (C) and the distributed strategy (D), $V_\Vert$ is substantially larger than $V_\perp$, which is the same pattern found in the experimental data.

### Joint angle variance: structure

**Fig. 6:**
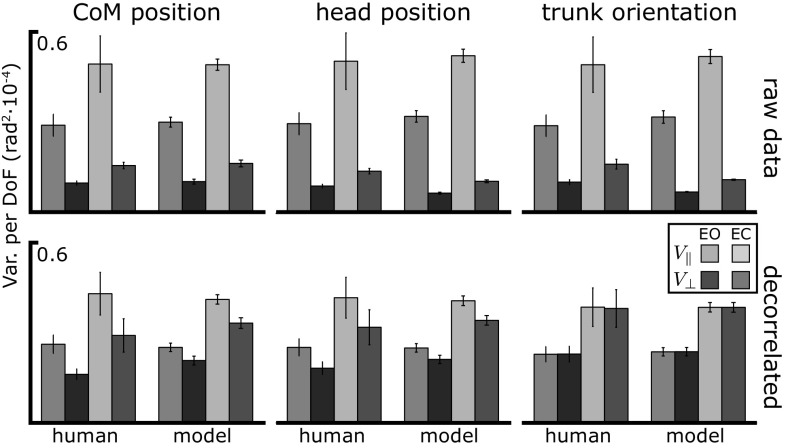
Mean variance per DoF of the joint angles over $30\,{\mathrm {s}}$ of quiet, upright stance, in the UCM space relative to CoM position, head position and trunk orientation as task variable. Experimental data are averages across subjects. The *top panel* shows the raw data, for the *bottom panel* the data was decorrelated. *Error bars* show the standard error

To analyze the covariance structure of the data generated by the model and compare it to the human data in more detail, we also performed a UCM analysis with respect to the head position and trunk orientation on the raw and the decorrelated data (see Sect. 3.3), for both the eyes open (EO) and eyes closed (EC) condition. Figure 6 shows the results for the human data and the distributed strategy with multi-joint coordination (D). The top panel shows the two components of variance along ($V_\Vert$) and orthogonal ($V_\perp$) to the corresponding UCM, comparing model with experimental data. Larger variance within the UCM than orthogonal to the UCM is observed under all conditions and for all three task variables in the experimental data. This means that there is substantially more sway in directions that leave the CoM, head position or trunk orientation invariant than in directions that do affect these task variables. The model simulations reproduce this effect. When vision is removed, both components of variance increase in magnitude. This increase was statistically significant in the empirical data (see Table 2) and is also reproduced by the model.

**Table 2:** ANOVA results for EO versus EC

EO versus EC		*p*-value	*F*
Head position	$V_\Vert$	${<} 0.001$	18.24
	$V_\perp$	${<} 0.001$	54.65
CoM position	$V_\Vert$	${<} 0.001$	18.48
	$V_\perp$	${<} 0.001$	54.59
Trunk orientation	$V_\Vert$	${<} 0.001$	18.18
	$V_\perp$	${<} 0.001$	43.96

The results for the decorrelated data are shown in the bottom panel of Fig. 6. The difference between the two components of variance is strongly reduced. A small UCM effect is still present in the decorrelated data set for CoM and head position, indicating that a portion of the difference in variance between the two subspaces is due to differences in variability of the underlying variables rather than to their covariation. Again, the model matches the data closely. For trunk orientation, the UCM effect disappears completely in the decorrelated data in both theory and experiment. This is a necessary effect, which is due to the properties of trunk orientation as a task variable (Verrel 2011).

### Joint angle variance: magnitude

**Fig. 7:**
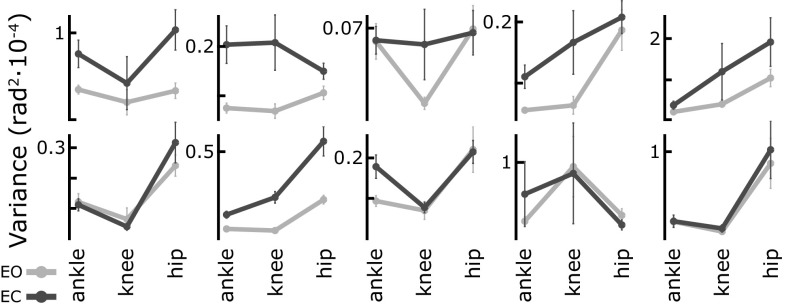
Average joint angle variance for ten individual experimental subjects, in EO and EC conditions. *Error bars* show the standard error

How does the model capture overall variance in joint space? While single subjects can have highly reproducible personal movement patterns, the inter-subject variability between these patterns is often much larger. Rather than trying to fit an “average participant,” we first need to analyze the variance within the population empirically. For each of the ten experimental subjects, Fig. 7 shows the average magnitude of the variance of ankle, knee and hip angles over $30\,{\mathrm {s}}$ of quiet stance ($N=24$ episodes per subject, error bars show the standard error). For each participant, variance is shown for the eyes open (EO) and eyes closed (EC) conditions. Clearly, the distribution of variance across the three joints is very diverse across participants. Within a subject, however, the pattern of joint variance is more coherent across the two conditions. It seems that each participant has a characteristic pattern of joint variance.

To analyze these subject-specific patterns statistically, we first normalized the joint excursion variance data using a Box–Cox transformation (Sakia 1992). Then we fitted a repeated measures model to the normalized data, using the function fitrm in the MATLAB Statistic and Machine Learning Toolbox. On this repeated measures model, we performed a two-way MANOVA with subject and condition as factors. This revealed a significant effect of subject ($$F = 18.0; p < 0.0001$$) and condition ($$F = 12.4; p < 0.0001)$$. Note that we can analyze subject as a factor here because there are multiple ($$N=24$$) observations for each subject, whereas in standard applications there is only one observation per subject.

But is this effect due to the differences in the shape of each subject’s variance pattern, or due to the differences in magnitude between the subjects? To answer this question, we transformed the data into independent components representing the shape and the magnitude of the joint angle variance. The magnitude was defined as the length of the joint angle variance vector. The shape was represented by the stereographic projection of the remaining unit length vector onto a plane. Again, we normalized the data using the Box–Cox transform and fitted repeated measures models and then performed a two-way MANOVA on the shape and a two-way ANOVA on the magnitude variable. For magnitude, both subject ($F = 32.2; p < 0.0001$) and condition ($F = 23.0; p < 0.0001$) showed significant effects. For the shape variable, however, subject showed a significant effect ($F = 22.0; p < 0.0001$), while condition did not ($F = 1.35; p = 0.25$).

**Fig. 8:**
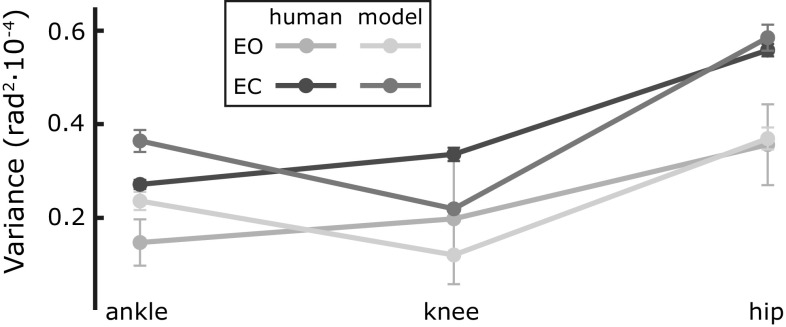
Joint angle variance for the model simulation and the experimental data (average across subjects), in EO and EC conditions. *Error bars* show the standard error

The effect of vision is thus primarily a change in the overall magnitude of sway. When vision is removed, the overall magnitude of the sway increases, while the relative amount of motion in each joint remains the same. The model captures this effect, as illustrated in Fig. 8, showing the average joint angle variance of the simulation data in both EO and EC conditions. To compare the magnitude, the average across subjects for the experimental data is also shown.

### Falls when a feedback loop is severed

To gain better understanding of the nature of the control problem, we opened the control loop in two different ways and observed the resulting fall trajectories. The first way was to remove the descending motor commands, setting $\dot{\lambda }(t) = 0$, while leaving the low-level spinal reflexes intact (compare Fig. 1). The second way was to make the motoneural activation, *E*, constant except for noise, effectively removing all control, but leaving the passive properties of the muscle–tendon systems intact.

**Fig. 9:**
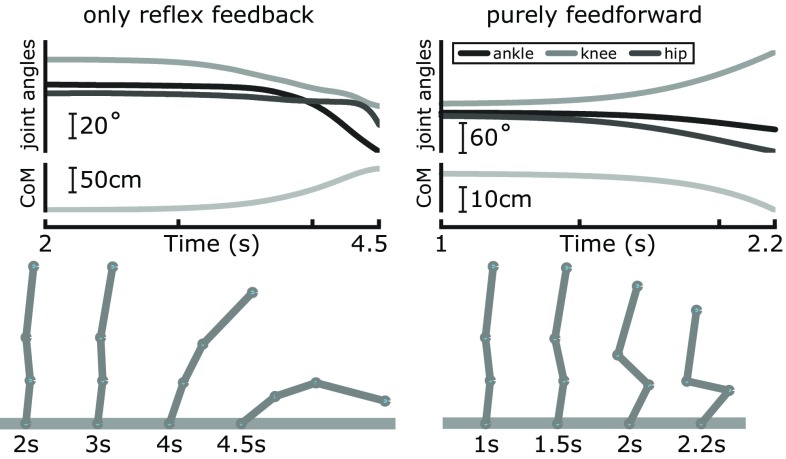
Effects of removing the feedback dynamics. Data are from single trials with the outer feedback loop severed, i.e., only the reflexive feedback left intact (*left*), and with purely feedforward control, i.e., all feedback removed (*right*). The top panels show the trajectories of the joint angles and the CoM until balance has clearly been lost. The *bottom panels* illustrate this by showing the sequential change of body configurations

Both alterations resulted in falls in the model simulations. The left side of Fig. 9 illustrates the effect of opening the outer feedback loop. At first, the body is partly stabilized by the spinal feedback and does not move much. The gains of the spinal feedback loops are not sufficient, however, to counter the destabilizing effects of gravity. After about 3 s, the body starts falling forward. All joint angles increase under the gravitational pull, until the body hits the floor. The center of mass shows the same movement pattern as the joint angles.^2^

The right side of Fig. 9 shows a simulation in which the spinal reflex feedback loop was opened. Instead of toppling over at the ankle with all joint angles decreasing, the body folds: The knee angle starts to increase, while ankle and hip angle decrease, resulting in a folding movement. The center of mass movement is much smaller than when only the outer feedback loop is severed. Clearly, without spinal feedback, the muscles do not react to motor commands in a meaningful way and thus do not resist the pull of gravity.

These two distinct patterns of falling are systematic in the model—with purely feedforward muscle activation, the body folds and collapses. With intact spinal reflex loops but purely feedforward descending commands, the body remains stretched, but topples over in anterior or posterior direction at the ankle, the joint with the lowest passive stiffness. To examine this phenomenon in detail, we simulated $N=1000$ trials in both conditions. For each trial, we tracked over time whether the body was still stretched or was folding. We defined a state as “folding” when at least two joints had changed in different directions relative to the initial condition and as “toppling” otherwise. The results are summarized in Table 3. Either the outer or both the outer and the spinal reflex loop were severed. Without any feedback, the number of toppling trials is essentially zero at any time, except for a transient state at the very beginning for a small number of trials. With spinal reflexes but no outer feedback loop, the 1st s is still relatively stable, most movement patterns are folding. As the time progresses, however, the folding patterns disappear and the number of toppling trials approaches 100%. These results highlight the functional role of the two neural feedback loops. The faster spinal reflexes stabilize against folding, the slower outer feedback loop stabilizes against toppling.

**Table 3:** Effects of severing the feedback loops

Condition	% Of toppling trials after			
	1 s	2 s	3 s	4 s
Outer loop cut	16.5	85.3	98.4	99.9
Inner loop cut	0.1	0	0	0

The numbers give the relative amount of trials where the body had started to “topple,” i.e., fall forward or backward as a whole, instead of falling straight down by “folding” at the hip and knee joints

## Discussion

We have developed a model that combines existing models of body biomechanics and muscle neurophysiology with novel ideas of how principles of neural control integrate these to generate movement behavior. The result is an account for how, depending on sensory information, muscle activation is generated in time, based on the idea of a compliant spring whose operating point is moved around in a throw-and-catch pattern (Lakie et al. 2003). The control law generates motor commands that are coordinated across multiple degrees of freedom (Martin et al. 2009). The resulting process model not only accounts quantitatively for experimentally observed data but also explains the function of the postulated mechanisms. A computational implementation of the model can actually stabilize upright stance in a body with multiple degrees of freedom, realistic biomechanics and muscle models based on realistic models of available sensory information.

### The origin of the UCM structure

The random fluctuations of the ankle, knee and hip joints during postural sway are not independent. The UCM analysis reveals that the point cloud of the data in joint space is elongated in directions that leave important task variables invariant and condensed in directions that do affect these variables. Removing the temporal correlation from the joint trajectories strongly reduces this difference in variance between the UCM and the orthogonal components (see Fig. 6). This implies that a large part of the difference in variance across the two subspaces comes from coordination across joints rather than from intrinsic differences in variance across different subspaces. The trunk orientation is a special case here, because any (non-random) differences $V_\Vert$ and $V_\perp$ for this task variable *must* be due to systematic covariation between the joints (Verrel 2011).

How does the structure of variance in joint space arise? One possibility would be that covariation is induced by the biomechanical coupling of the limbs. Such coupling may be characterized through the modes of oscillation that arise in multi-segment pendula (Alexandrov et al. 2005). The two modes differ in how easily they can be excited. Because sway in the ankle strategy mode moves the whole body, it takes larger amounts of force to overcome the larger inertia, while sway in the hip strategy mode accelerates smaller portions of the body mass and thus requires less force. Random fluctuations from, e.g., noise in the motor system, are thus expected to generate more sway in the hip mode than in the ankle mode. Because hip mode sway moves the CoM less than ankle model sway, this would result in more variance along the UCM than orthogonal to it, as observed. The results of our simulation study show, however, that the control hypotheses based on co-contraction or purely local control result in sway patterns with a covariance structure opposite of what is experimentally observed in humans. The biomechanical properties of the simulated body are, of course, the same for all control hypotheses, so these alone cannot constitute the defining factor for the shape of the variance structure.

The essential factor in generating the correct multi-joint sway patterns appears to be the active coordination between joints: the neural activation matrix *R*, the muscle distribution matrix *A*, the inertia matrix *M* and the Jacobian matrices $J_p$ and $J_o$ (Eqs. 23, 18 and 21). Surprisingly, the details of the transformation from task to joint level, represented by the Jacobian matrices, do not have a large effect on the variance structure. Figure 5 shows that the ankle strategy with multi-joint coordination, using the reduced Jacobian $\tilde{J}_p$, results in multi-joint sway patterns that are very similar to both the distributed strategy, which uses the full Jacobian, and the experimental data. This indicates that the essential components for generating the correct sway patterns are the coordination terms *R*, *A* and *M* on the joint level.

This finding is at odds with the optimal control approach to motor control (Wolpert 1997; Todorov and Jordan 2002), in that the optimality of the controller does not appear to matter much. One of the control hypotheses we examined, the distributed strategy with multi-joint coordination, is optimal in the sense that it generates the desired task feedback to stabilize the body in space with the smallest possible changes in joint angles. The joint space changes generated by the ankle strategy with multi-joint coordination, in contrast, are larger. The structure of the covariance generated by both patterns is, however, very comparable. This result supports the notion that it is overall more beneficial for biological systems to settle for “good-enough” solutions to a motor task, rather than optimal, because the large required effort is usually not worth the small additional gain (Loeb 2012).

An interesting factor in this discussion is the presence of highly individual movement patterns, as shown in Fig. 7. This could mean that different individuals have settled on good-enough movement strategies at some point, that are then preserved over time without a strong drive for further optimization. It is also possible, however, that these patterns correspond to local minima in the cost landscape, where small changes in any directions increase cost, discouraging change and prohibiting further adaptation. It is even conceivable that each of these patterns is actually optimal for the specific body configuration of each individual. Deciding between these possibilities would require fixing a cost function and optimizing the parameters for different body types.

It is important to point out that although our results are at odds with the formalism of optimal control, they do resonate positively with the principle of minimal intervention, which states that only deviations that interfere with the task at hand should be corrected, and no effort should be spent to correct deviations that do not affect the task (Todorov 2004). In our model, deviations that do not affect the task variable would generate no feedback on the body-in-space level and thus no descending motor commands.

### Control strategies

How do the control hypotheses explored here relate to those discussed in the postural literature? The ankle and hip strategies have been identified as two distinct modes of control within a potential continuum of movement patterns. This was based on the response to support surface translations (Horak and Nashner 1986). Creath and colleagues found that signatures of these two patterns are visible also in quiet stance (Creath et al. 2005). Still, the quantitative predominance of the ankle strategy during quiet stance has justified the approximation of a single-link inverted pendulum in most mathematical models and experimental designs. Winter gave a formal justification for this approximation by pointing out that the high degree of correlation between the difference of the whole-body center of pressure and the ${\mathrm {CoM}}$ and the acceleration of the CoM are predicted by the single-DoF inverted pendulum model (Winter 1995).

While this argument is sound at the level of kinematics, extending it to muscle forces is problematic. Due to the inertial coupling between the different body segments, a muscle contraction that generates torque at the ankle will also accelerate all other joints along the body (Zajac and Gordon 1989). To generate movement only at the ankle and keep the other joints still, these interaction torques at the knee and hip have to be canceled out by active forces from muscles spanning these joints. Indeed, Horak and colleagues reported distinct activation patterns of hamstrings and paraspinal muscles during ankle strategy movements (Horak and Nashner 1986). A recent detailed study revealed that as much as 70% of the joint torques cancel out interaction torques from other joints (Sasagawa et al. 2014). A more refined characterization of the two strategies would thus distinguish between kinematic and kinetic variants. For instance, a purely kinematic ankle strategy would be one in which active movement is generated only at the ankle, while all torques generated around other joints are strictly aimed at canceling out interaction torques. In this light, the observation that 30% of hip torques *cannot* be accounted for by interaction torques suggests either imperfect control or a mixed strategy, in which muscles acting on the hip and knee joints are actively recruited to generate movement at these joints independent of the ankle joint.

We have implemented such a kinematic version of the ankle strategy that generates active movement at the ankle, but applies coordinated torques at the knee and hip joints to account for inertial interaction torques, and shown that the patterns of multi-joint sway generated in simulations are very similar to those in experimental data (see Sect. 4.2). The kinetic ankle strategies, on the other hand, that feed sensor information exclusively into ankle torque, while stabilizing the other joints locally and lacking coordination, result in strikingly different sway patterns (Fig. 5). This result supports the notion that active coordination between the different joints plays an important role in structuring joint variance in quiet, upright stance.

### Standing upright

We have shown that a postural control system with two nested feedback loops can account for the stability of multi-segment upright posture. The inner loop stabilizes the muscle–joint systems for each joint angle locally, up to a certain degree. It is not strong enough to counteract the destabilizing effect of gravity, especially at the ankle joint. The velocity of the resulting falls is reduced, though, to a degree where the slow outer feedback loop can act to stabilize the body in space.

The outer feedback loop resists falling by generating motor commands that drive the body backward when the sensed body velocity is consistent with falling forward and, conversely, generating motor commands that drive the body forward when a backward fall is sensed. Failure of this outer feedback loop leads to the whole body falling either forward or backwards at the ankle, depending on the initial body velocity or acceleration. Falls involving substantial movement in all joints with the CoM moving mostly downwards are prevented by the inner loop and not observed in simulations when it is intact.

## Conclusion

In conclusion, three key ingredients of our model help understand multi-segment postural control: (1) The inner feedback loop of each muscle–joint system enables each degree of freedom to counteract the mechanical effect of gravitational load, preventing collapse. (2) The outer feedback loop about body in space stabilizes postural sway against falling. Accounting for kinetics by weighing the forces at each joint according to inertial coupling forces from all body segments acting on each joint appears to be a critical factor for the success of control schemes *C* and *D* versus *A* and *B*. (3) The UCM structure of variance reflects a kinematic control law in which deviations in joint space that move the body in space are selectively counteracted, while deviations not affecting the body in space are not. The distribution of the control signal across joints by the combined kinetic and kinematic control laws induces the covariation among joints that is observed as the UCM structure of variance.

## Footnotes

1 Note that this is a harmonic oscillator in the state variable $v = \dot{p}$, so the acceleration of the state variable is $\ddot{v} = \dddot{p}$.

2 Note that the final phase of the fall is not realistic as the heel would lift off the floor at some point. This was not modeled.
